# Serotyping and Antimicrobial Susceptibility Profiling of *Glaesserella parasuis* Isolated from Diseased Swine in Brazil

**DOI:** 10.3390/pathogens11121443

**Published:** 2022-11-30

**Authors:** Givago Faria Ribeiro Silva, Luisa Zanolli Moreno, Carlos Emílio Cabrera Matajira, Ana Paula Santos Silva, Kawany Miyazaki Araújo, Vasco Túlio Moura Gomes, Mikaela Renata Funada Barbosa, Maria Inês Zanolli Sato, Andrea Micke Moreno

**Affiliations:** 1 Department of Preventive Veterinary Medicine and Animal Health, School of Veterinary Medicine and Animal Science, University of São Paulo, Av Prof Dr Orlando Marques de Paiva, 87, São Paulo 05508-270, SP, Brazil; 2 Phibro Animal Health Corporation–Av. Pres. Tancredo de Almeida Neves, 1063, São Paulo 071112-070, SP, Brazil; 3 Facultad de Ciencias Básicas, Universidad Santiago de Cali, Cali Calle 5 #62-00, Vale do Cauca, Colombia; 4 Environmental Company of the State of São Paulo (CETESB), Av. Prof. Frederico Hermann Júnior 345, São Paulo 05459-900, SP, Brazil

**Keywords:** *Glaesserella parasuis*, antimicrobial resistance, serotype, pig, Glasser’s disease, PCR, MIC

## Abstract

*Glaesserella parasuis* is one of the major pathogens in swine intensive production systems. To date, 15 serovars have been described, and the prevalence of these serotypes in different geographical regions has been identified by several methods. *G. parasuis* outbreaks could be controlled with vaccination if it were not for serovar diversity and limited cross-serovar protection; consequently, antibiotic therapy continues to be necessary for infection control. Here, we present the isolation, identification, serotyping, and antibiotic susceptibility profiling of *G. parasuis* from diseased swine in Brazil. A total of 105 *G. parasuis* strains, originating from nine different Brazilian states, were evaluated, and serotypes 4 and 5 were found to be the most prevalent (27.6% and 24.8% respectively). Aminoglycosides, florfenicol, tiamulin, and β-lactams were tested, and they presented lower resistant rates against *G. parasuis* strains. The highest resistance rates were observed against tylosin (97.1%), sulfadimethoxine (89.5%), danofloxacin (80%), trimethoprim/sulfamethoxazole (62.5%), enrofloxacin (54.3%), and clindamycin (50.5%). Multidrug resistance was detected in 89.5% of tested strains, and a total of sixty resistance profiles were identified. The cluster analysis of resistance patterns showed no correlation with the isolation year or *G. parasuis* serotype.

## 1. Introduction

Pigs can be colonized by different microorganisms before weaning, but some of those agents are potentially pathogens, such as *G. parasuis*. Infection caused by this agent has a serious impact on pig production. Therefore, the correct diagnosis of this disease is essential to establish appropriate control measures. Fibrinous polyserositis and arthritis caused by *G. parasuis* (Glasser’s disease) is usually diagnosed based on herd history, clinical signs, necropsy findings, and bacterial isolation [1].

*G. parasuis* is a Gram-negative, NAD-dependent, and fastidious bacterium, and its isolation in pure culture from diseased animals is usually difficult, particularly if the animals received antimicrobial treatments before sample collection. The isolation of the microorganism is important, not only to genotyping and serotyping but also for antimicrobial sensitivity determination, which are very useful for disease control. The isolation of the microorganism is important, not only to genotyping, virulence studies, and serotyping but also for antimicrobial sensitivity determination, which are also very useful for disease control. 

The impact of Glässer’s disease can be reduced in some herds by the use of vaccination with commercial bacterins based on serotype and autogenous vaccines; however, the great serovar diversity and the limited cross-serovar protection still represent an important challenge for disease control. The evaluation of virulence factors associated with infection by *G. parasuis* and the identification of epitopes with good antigenicity are extremely important for the development of subunit vaccines able to block the early steps of infection and reduce the dissemination of pathogens in internal organs. For this reason, antibiotic therapy continues to be necessary for the management of Glässer´s disease outbreaks [1,2]. 

For *G. parasuis* control, β-lactams, phenicols, macrolides, sulphonamides, and tetracyclines are often recommended [3]. However, resistance to sulphonamides, tetracycline, quinolones, and florfenicol has already been described for *G. parasuis* among Chinese, Australian, Brazilian, and Czech herds [4,5,6,7], and in the Spanish swine herds, multidrug-resistant *G. parasuis* strains were described causing infection [8]. Here, we present the isolation, identification, serotyping, and antibiotic susceptibility profiling of a collection of *G. parasuis* isolated from diseased swine in Brazil.

## 2. Materials and Methods

### 2.1. Samples and Bacterial Isolation

**Figure 1:**
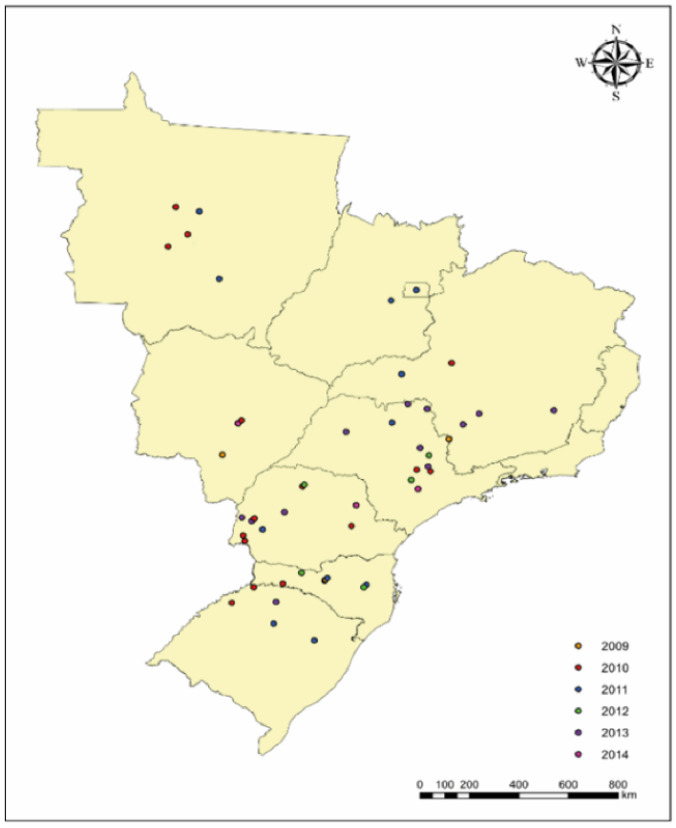
Distribution of evaluated herds according to Brazilian states were strains of *G. parasuis* that were isolated.

A collection of 105 *G. parasuis* strains isolated from 82 animals raised in 49 herds was evaluated. Studied herds were in the following Brazilian states: Mato Grosso, Mato Grosso do Sul, Minas Gerais, Paraná, Rio Grande do Sul, Santa Catarina, Distrito Federal, and São Paulo. These strains are part of the Swine Health Laboratory (FMVZ-USP) bacterial collection (Figure 1).

Different tissue and fluid samples (lung, brain, and pericardial and thoracic fluid) were plated on blood agar (5% defibrinated sheep blood) with a streak of *Staphylococcus aureus* and incubated at 37 °C for 48 h with 5% CO_2_. Presumptive *G. parasuis* isolates were cultured in BHI—brain–heart infusion broth (Difco, Sparks, MD, USA) with 5% of bovine fetal serum and 10 μg/mL of NAD (nicotinamide adenine dinucleotide, Sigma, St. Louis, MO, USA) for the proposed analysis.

### 2.2. G. parasuis Identification

*G. parasuis* strains were selected based on the species-specific amplification of the 16S rRNA gene, as previously described [9], and after reactivation and culture the results were confirmed by MALDI-TOF MS (matrix-associated laser desorption-ionization–time of flight mass spectrometry) (Bruker Daltonics, Inc. Billerica, MA, USA). Isolates were also tested for the amplification of the group 1 vtaA gene [10] that has been associated with virulent strains of *G. parasuis*.

### 2.3. Serotyping

Serotyping was performed by multiplex PCR assay according to a protocol described previously [11]. The multiplex PCR was carried out into two separated reactions to enable a better distinction among products of similar lengths. The first reaction included the primers for identification of serovars 2, 3, 6, 7, 9, 10, and 11, and the second reaction included primers for serovars 1, 4, 5, 8, 12, 13, 14, and 15. PCR was performed with HOT FIREPol^®^ Taq DNA Polymerase (Solis BioDyne, Tartu, Estonia), to a total volume reaction of 50 μL, considering a final concentration of 2 mM MgCl_2_, 200 μM of each dNTP, and 0.2 μM of each primer. The Bio-Rad T1000 cycler (Bio-Rad Laboratories, Hercules, CA, USA) thermocycler conditions were 15 min at 95 °C, 34 cycles of 94 °C for 45 s, 58 °C for 60 s, and 72 °C for 60 s, followed by a final extension step at 72 °C for 10 min.

### 2.4. Antibiotic Susceptibility Profiling

The MIC (minimal inhibitory concentration) was determined using the broth microdilution technique following the description by Prüller et al. [12] and Clinical and Laboratory Standards Institute recommendations [13]. The ready-made microplates containing the antimicrobials, Sensititre™ Bovine/Porcine BOPO6F (TREK Diagnostic Systems/Thermo Fisher Scientific, Waltham, MA, USA), were used to evaluate the strains. Staphylococcus aureus ATCC 29,213 and Streptococcus pneumoniae ATCC 49,619 were tested as the quality controls. The values of MIC50 and MIC90 for all antimicrobials were determined as described by Schwarz et al. [14]. Strains were classified as multidrug-resistant when they showed resistance to three or more classes of antimicrobials [15]. 

The breakpoints applied for the results description were obtained mainly in the CLSI [13] and are described in Table 1. When the breakpoints values were not present in VET01S [13], the CLSI performance standard M100, 32nd edition [16] and literature [17,18] were used. The breakpoints were selected on the basis of the following criteria: the first choice were the values described for swine respiratory diseases, and if there were no recommendations in the CLSI or EUCAST, the literature references were used. The analysis of resistance profiles was conducted with software Bionumerics 7.6 (Applied Maths NV, Saint-Martens-Latem, Belgium); profiles were evaluated as categorical data using the Ward method and different value coefficients. A minimum spanning tree (MST) was plotted to visualize the relationships between the identified resistance profiles. The association analysis between susceptibility profiles and the isolation date was carried out using Fisher’s exact test and SPSS 16.0 software (SPSS Inc., Chicago, IL, USA).

## 3. Results

*G. parasuis* strains were isolated from brain (1%–1/105), lung (91.4%–96/105), thoracic cavity (1.9%–2/105), and pericardium (5.7%–6/105) samples. In total, 35.2% of the strains were isolated, between 2009 and 2010, while the remaining 64.8% were obtained between 2011 and 2014, from nine Brazilian states (Figure 1). 

All strains were identified as *G. parasuis* by MALDI-TOF, and from PCR to the 16S rRNA gene, all were positive for group 1 vtaA gene detection. Among the 105 tested strains, serotypes 4 and 5 were the most prevalent (27.6% and 24.8%, respectively), followed by serotypes 13 (18.1%), 14 (13.3%), and 1 (11.4%) (Table 1). 

**Figure 2:**
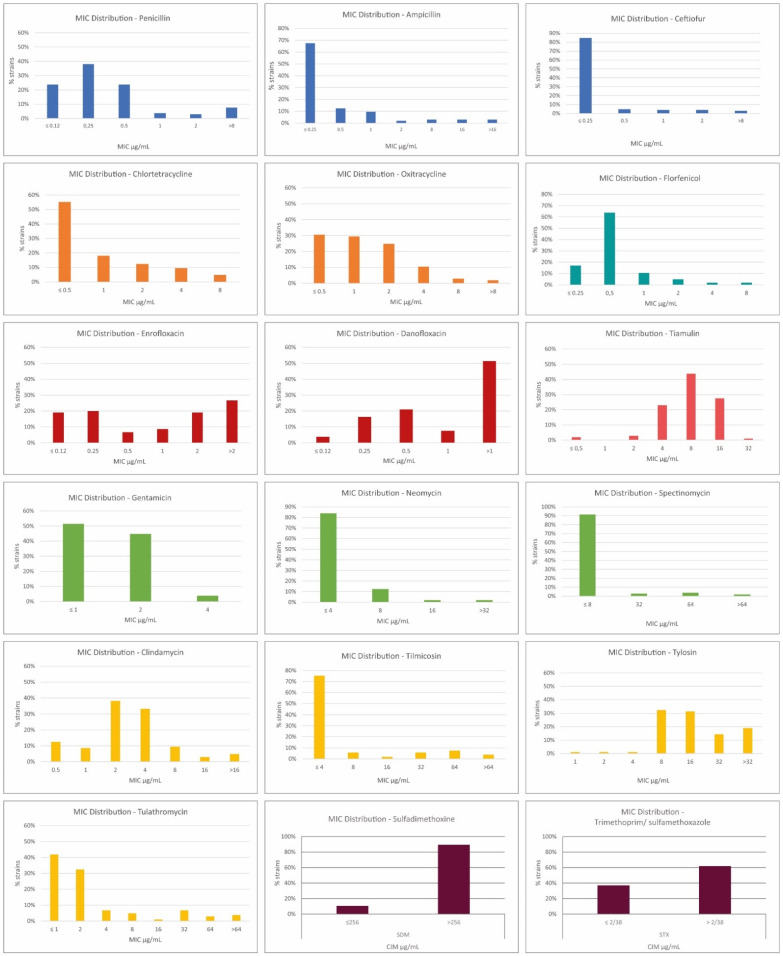
Distribution of MIC values of *G. parasuis* according to antimicrobial testing.

All 105 strains were susceptible to gentamycin (Table 2). The highest resistance rates were observed against tylosin, sulfonamides, fluoroquinolones, and clindamycin. The aminoglycosides presented a lower resistant rate against Brazilian *G. parasuis*, while the florfenicol, tiamulin, and β-lactams tested inhibited most of the strains studied. The distribution of MIC values is presented in Figure 2 and Table 3.

A total of 98.1% (103/105) of isolates were resistant to at least one of the tested antimicrobial classes; only two isolates were characterized by susceptibility to all tested drugs. Multidrug resistance was detected in 89.5% (94/105) of tested strains, with 23.8% (25/105) being resistant to more than five antibiotic classes (Table 4). No significant difference was observed between the susceptibility profiles and the isolation year (*p* value = 0.43).

**Figure 3:**
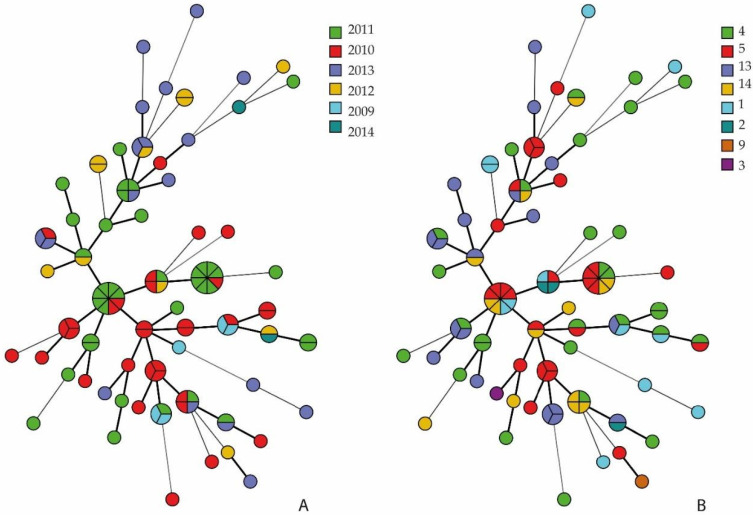
Minimum spanning tree (MST) showing the relationship among the *G. parasuis* resistance profiles. Single circles correspond to isolates presenting exclusive resistance profiles, while divided circles correspond to isolates presenting the same resistance profile. The distance between circles and the line width correspond to the similarity between the identified profiles. The highlighted color scheme corresponds to the year (**A**) and serotype (**B**) information.

No correlation between clusters according to resistance profiles and isolates’ origin and serotype was detected (Figure S1). The MSTs demonstrate the dispersion of *G. parasuis* serotypes among the resistance profiles obtained (Figure 3B); the dispersion of strains according to isolation date and resistance is observed in Figure 3A.

## 4. Discussion

The observed predominance of serotypes 4, 5, 13, and 14 corroborates previous Brazilian reports [19,20,21,22]. These serotypes are also predominant in European and Chinese swine herds, as previously described [8,23,24]. Despite not having a proven correlation, these serotypes have been associated with moderately and highly virulent *G. parasuis* strains [1], which agrees with all of the studied isolates being positive for group 1 vtaA gene amplification, which has also been associated with virulent *G. parasuis* [10].

The main issue for the evaluation and comparison of *G. parasuis* antimicrobial susceptibility results is the lack of specific standardized breakpoints. Most published studies apply respiratory disease breakpoints, when available, or make an approximation from specific *Actinobacillus pleuropneumoniae* and/or *Histophilus somni* breakpoints [5,7,8,25]. In the present study, breakpoints were selected with the following criteria: those described for swine respiratory diseases were preferably chosen in CLSI documents, and when there was no description in the CLSI or EUCAST, a literature reference was used.

Considering the resistance profiles identified, it was possible observe that the highest resistance rates were found against tylosin (97.1%), sulfadimethoxine (89.5%), danofloxacin (80%), trimethoprim/sulfamethoxazole (62.5%), enrofloxacin (54.3%), and clindamycin (50.5%). Resistance against tylosin and clindamycin are becoming very frequent in swine pathogens since these drugs were extensively used in the last 40 years as growth promoters (this method was banned in Brazil in 2020). *G. parasuis* presenting high MIC values against tylosin and clindamycin are also described by De la Fuente et al. [8] in Spain and Miani et al. [6] in Brazil. 

Resistance to sulphonamides observed in this study has been described in the literature, to varying degrees in different countries. In Denmark, Aarestrup et al. [26] reported that 3.8% of *G. parasuis* strains were resistant to trimethoprim/sulphamethoxazole. In Spain and the UK, de la Fuente et al. [8] reported that 53.3% of Spanish and 10% of British strains were resistant to trimethoprim/sulphamethoxazole. In the present study, a higher level of resistance to trimethoprim/sulphamethoxazole and sulfadimethoxine was observed. Zhao et al. [26] described the identification of both sul1 (6.29%) and sul2 (1.4%), in 143 *G. parasuis* strains from China and ranked these genes as most associated to trimethoprim/sulfamethoxazole resistance. 

Zhou et al. [7] reported a resistance rate to enrofloxacin of 70% (78/110), and Zhao et al. [26] described 55.9% (80/143), with both studies being conducted in China. These rates are similar to the results observed in our strains (54.3%). Strains evaluated by Zhao et al. [26] presented different gyrA and parC mutations, affecting 125 of 143 strains. The association between these mutations and fluoroquinolone resistance is largely described in Gram-negative bacteria and Gram-positive bacteria from pigs. 

Contrary to these observations, Miani et al. [6] described different results in Brazil, indicating that the fluoroquinolones class is a good choice in *G. parasuis* control. These authors reported a very low MIC90 against quinolones (MIC 90 = 0.25 µg/mL to enrofloxacin and 0.12 µg/mL to danofloxacin) when evaluated in 50 clinical isolates. Our findings suggest that this antimicrobial class must be avoided because of growing resistance rates (MIC 90 >2 µg/mL to enrofloxacin and >1 µg/mL to danofloxacin) and the importance to fluoroquinolones of controlling human pathogens. 

Resistance to the tetracycline drugs tested (oxytetracycline and chlortetracycline) was present in 40 and 26.7% of strains, respectively. The spread of the tetracycline class resistance in swine production is expected because it is one of the most used in-feed antimicrobials in preventive and metaphylactic programs in Brazil [27]. Strains of *G. parasuis* carrying tet B (23.78%) and tet C (3.5%) genes were described in China and are probably circulating in studied strains [26]. The MIC90 of both tetracyclines evaluated here was 4 µg/mL (considering 105 strains). Recent studies describe MIC90 of 8 µg/mL [6] in Brazil and MIC90 of 16 µg/mL in Germany [28].

Aminoglycosides resistance rates were zero against gentamicin, and MIC90 values were low against neomycin and spectinomycin in evaluated strains, but these drugs are not the most indicated to respiratory or systemic disease treatment in swine. The global results indicate variable MIC90 values of this class; Brogden et al. [28], in Germany, describe MIC90 of gentamicin of 4 µg/mL and neomycin of 16 µg/mL. The MIC90 of spectinomycin (≤8.0 µg/mL) was lower than described for Spanish and British strains [8], Chinese strains [7], and in Brazil [6].

Resistance rates and MIC90 values against b-lactams were low but looking at the different representative drugs tested it is possible to observe crescent resistance rates. These rates were lower than ceftiofur, followed by ampicillin, and higher than penicillin. These findings agree with values observed in British isolates and differ from Spanish strains, as described by De la Fuente et al. [8]. As described by Brogden et al. [28], the observation of bimodal or broad MIC distributions for several antimicrobial agents (Figure 2) indicates the presence of non-wild type isolates, with acquired mechanisms of resistance. The presence of β-lactamases, for example, is possible in isolates with elevated MICs of penicillin, ampicillin, and ceftiofur. Genes related to β-lactam resistance described in *G. parasuis* strains to date are blaTEM-1 and blaROB-1 [26].

Some macrolide compounds tested (tilmicosin and tulathromycin), pleuromutilin (tiamulin), and phenicol (florfenicol) showed low rates of resistance and low MIC90 values. These findings are quite interesting because these drugs are largely used for treating respiratory and enteric infectious diseases in Brazilian swine, especially tiamulin [27]. Florfenicol resistance was observed in 1.3% of tested strains; other studies described no resistance to this antimicrobial [7,8,28], unlike Miani et al. [6], who described 40% of florfenicol resistance. Zhao et al. [26] described 9% (13/143) of *G. parasuis* strains studied carrying gene floR, which was previously described in this bacterial species [29]. The emergence of florfenicol resistance in *G. parasuis* strains was related to a novel small plasmid pHPSF1 bearing floR. This plasmid is similar to other *Pasteurellaceae* plasmids, suggesting that these species prefer to exchange genetic elements with each other [26]. Chromosomal genomic islands, carrying 13 antimicrobial resistance genes, were described in *G. parasuis* strains from China. These islands harbored resistance genes for tetracyclines [tet(B)], β-lactams (blaROB-1), sulphonamides (sul2), chloramphenicol (catIII), and aminoglycosides [aph(300)-Ib, aph(6)-Id, and aph(30)-Ia]. These results highlight the important roles that mobile elements play in capturing and diffusing antibiotic resistance genes between plasmids and chromosomes in *Pasteurellaceae* [30]. 

Antimicrobial resistance is currently one of the world’s biggest concerns in terms of animal and public health. The world organization for animal health (OIE) standards provide global recommendations for controlling antimicrobial resistance, including lists of antimicrobial agents of veterinary importance to treat animal diseases. In parallel, the world health organization (WHO) has also developed a list of critically important antimicrobial agents in human medicine [31]. Our data indicate that multidrug resistance is present in 89.5% of Brazilian *G. parasuis* strains tested. Antimicrobial use as a preventive, metaphylactic, and therapeutic treatment is certainly contributing to the selection of these strains. It is particularly concerning that, for example, three out of six antimicrobials, for which resistance was described here in more than 50% of strains, are categorized by the WHO as critically important antimicrobials with maximum priority. 

Excessive antimicrobial use around the time of colonization of the upper respiratory tract can interfere with the colonization by *G. parasuis,* and this interference is not limited to this species but also influences the rest of the microbiota. The reduction in bacterial diversity in microbiota in piglets can cause the poorer performance of the immune system, as previously described [20,31,32]. 

The use of strategic antimicrobial treatments may only be advised in a few limited situations, mainly to treat piglets during a disease outbreak, which is important not only for health but also for welfare issues. Alternative control measures should be taken to minimize the potential increase in Glässer’s disease cases caused by resistant *G. parasuis* [31]. Therefore, providing veterinarians with data on the prevalent serotypes and on the resistance profiles of the members of this bacterial species is an effective way of contributing to the correct choice of antimicrobials.

## Figures and Tables

**Table 1:** MIC range observed, and breakpoints applied to *G. parasuis*.

Antimicrobial	MIC Range (µg/mL)	MIC Breakpoints		
		Susceptible	Intermediary	Resistant
Penicillin	≤0.12–2.0	≤0.25	0.5	≥1.0
Ampicillin	≤0.25–1.0	≤0.5	1.0	≥2.0
Ceftiofur	≤0.25–8.0	≤2.0	4.0	≥8.0
Chlortetracycline	≤0.5–8.0	≤0.5	1.0	≥2.0
Oxytetracycline	≤0.5–8.0	≤0.5	1.0	≥2.0
Danofloxacin	≤0.12–1.0	≤0.25	-	-
Enrofloxacin	≤0.12–2.0	≤0.25	0.5	≥1.0
Florfenicol	≤0.25–8.0	≤2.0	4.0	≥8.0
Spectinomycin	≤8.0–64.0	≤32.0	64	≥128
Gentamicin	≤1.0–16.0	≤2.0	4.0	≥8.0
Neomycin	≤4.0–32.0	≤8.0	-	-
Sulfadimethoxine	256	≤256	-	>256.0
Trimethoprim/sulfamethoxazole	2/38	≤2/38	-	≥4/76
Clindamycin	≤0.25–16.0	≤0.5	1.0–2.0	≥4.0
Tylosin	≤0.5–32.0	≤1.0	2.0–4.0	˃4.0
Tilmicosin	≤4.0–64.0	≤16.0	-	≥32.0
Tulathromycin	≤1.0–64.0	≤16	32	≥64.0
Tiamulin	≤0.5–32.0	≤16.0	-	≥32.0

**Table 2:** *Glaesserella parasuis* distribution according to identified serotype and year of isolation—N (%).

Year	Serotype								Total
	1	2	3	4	5	9	13	14	
2009	1 (8.3)	0	0	1 (3.4)	0	0	3 (15.8)	0	5 (4.8)
2010	1 (8.3)	2 (66.7)	0	11 (37.9)	7 (26.9)	0	5 (26.3)	6 (42.9)	32 (30.5)
2011	3 (25.0)	0	0	10 (34.5)	12 (46.2)	0	6 (31.6)	6 (42.9)	37 (35.2)
2012	4 (33.3)	0	0	2 (6.9)	3 (11.5)	0	1 (5.3)	1 (7.1)	11 (10.5)
2013	3 (25.0)	1 (33.3)	1 (100)	3 (10.3)	4 (15.4)	1 (100)	4 (21.1)	1 (7.1)	18 (17.1)
2014	0	0	0	2 (6.9)	0	0	0	0	2 (1.9)
Total	12 (100)	3 (100)	1 (100)	29 (100)	26 (100)	1 (100)	19 (100)	14 (100)	105 (100)

**Table 3:** Distribution of MIC values, MIC50, MIC90, and resistance rates identified in *G. parasuis* strains.

Antimicrobial	N° of Isolates with MIC of (µg/mL)											MIC50 (µg/mL)	MIC90 (µg/mL)	Resistance N (%)
	0.12	0.25	0.5	1	2	4	8	16	32	64	128			
Penicillin	25	40	25	4	3	0	0	8				≤0.25	1.0	15 (14.3)
Ampicillin		71	13	10	2	0	3	3	3			≤0.25	1.0	11 (10.5)
Ceftiofur		89	5	4	4	0	0	3				≤0.25	0.5	3 (2.9)
Chlortetracycline			58	19	13	10	5					≤0.5	4.0	28 (26.7)
Oxytetracycline			32	31	26	11	3	2				1.0	4.0	42 (40.0)
Danofloxacin	4	17	22	8	54							>1.0	>1.0	84 (80.0)
Enrofloxacin	20	21	7	9	20	28						1.0	>2.0	57 (54.3)
Florfenicol		18	67	11	5	2	2					0.5	1.0	2 (1.9)
Spectinomycin							96	3	0	4	2	≤8.0	≤8.0	2 (1.9)
Gentamicin				54	47	4	0	0				1.0	2.0	0 (0)
Neomycin						88	13	2	0	2		≤4.0	8.0	4 (3.8)
Clindamycin		0	3	9	40	35	10	3	5			2.0	8.0	53 (50.5)
Tylosin			0	1	1	1	34	33	15	20		16	>32.0	102 (97.1)
Tilmicosin						79	6	2	6	8	4	≤4.0	64.0	18 (17.1)
Tulathromycin				44	34	7	5	1	7	3	4	2.0	32.0	7(6.7)
Tiamulin			2	0	3	24	46	29	1			8.0	16.0	1 (1.0)
**Antimicrobial**	**N° of Isolates with MIC of (µg/mL)**											**MIC50 (µg/mL)**	**MIC90 (µg/mL)**	**Resistance %**
Sulfadimethoxine	≤256					>256								
	11					94						>256	>256	89.5
	**N° of Isolates with MIC of (µg/mL)**											**MIC50**	**MIC90**	**Resistance**
Trimethoprim/sulfamethoxazole	≤2/38					>2/38						**(µg/mL)**	**(µg/mL)**	**%**
	39					66						>2/38	>2/38	62.9

**Table 4:** Frequency of *G. parasuis* strains presenting multidrug resistance to antimicrobials tested.

Resistance Profiles	N	(%)
Susceptible	2	1.9
≤2 antimicrobial classes	9	8.6
3 antimicrobial classes	27	27.7
4 antimicrobial classes	42	40.0
5 antimicrobial classes	18	17.1
6 antimicrobial classes	4	3.8
7 antimicrobial classes	3	2.9
Total	105	100.0

## Data Availability

The data sets and materials are available from the corresponding author on reasonable request.

## Supplementary Materials

The following supporting information can be downloaded at https://www.mdpi.com/article/10.3390/pathogens11121443/s1, Figure S1: Dendrogram showing the relationship among the *G. parasuis* resistance profiles, isolation site, origin, year, and serotype.
