# Supplementary figures and images for: Serotyping and Antimicrobial Susceptibility Profiling of Glaesserella parasuis Isolated from Diseased Swine in Brazil
Source: Pathogens. 2022 Nov 30;11(12):1443. doi: 10.3390/pathogens11121443 (PMC9785225; doi:10.3390/pathogens11121443)

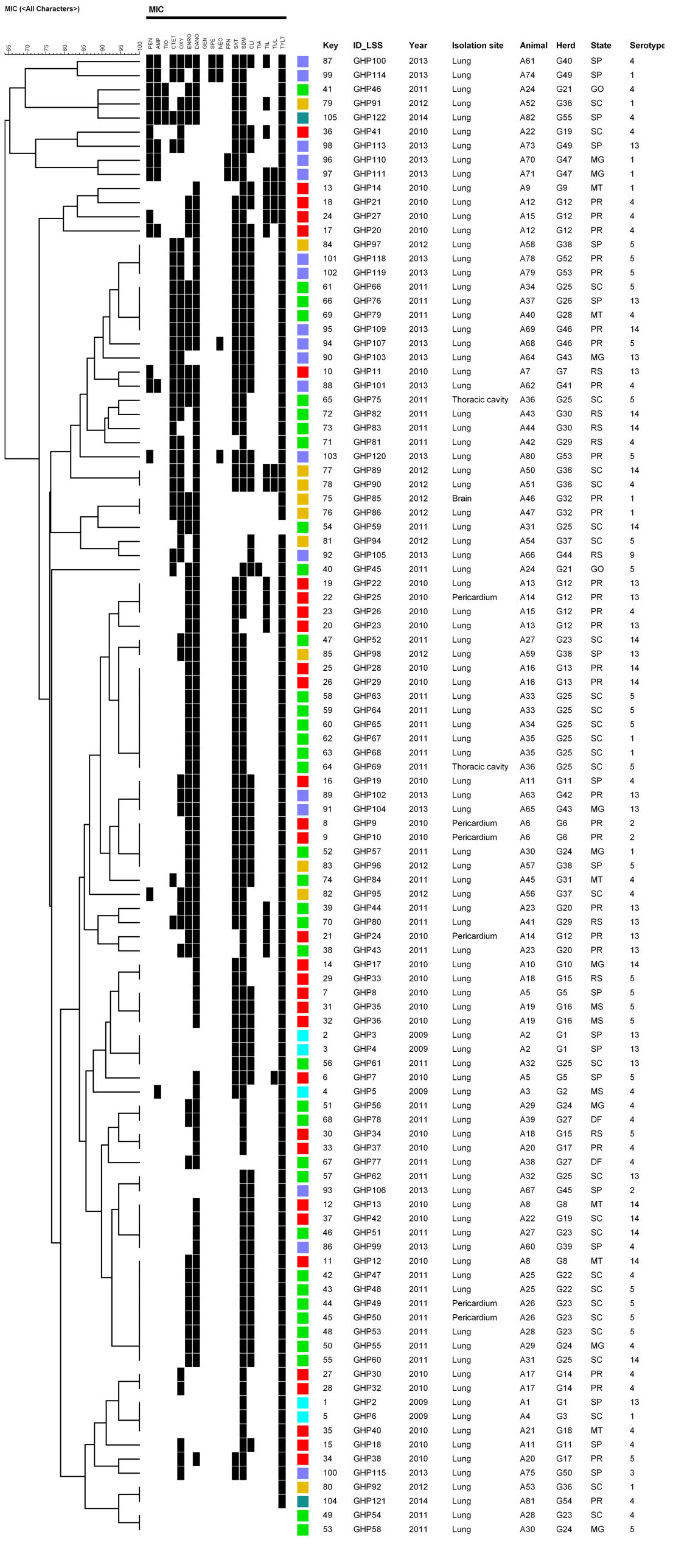

Supplement: Supplementary file 1 [file pathogens-11-01443-s001.zip › pathogens-2001070-supplementary.jpg]
